# Tetra­kis(1,3-diphenyl­propane-1,3-dionato)hafnium(IV)

**DOI:** 10.1107/S1600536810030400

**Published:** 2010-08-04

**Authors:** J. Augustinus Viljoen, Hendrik G. Visser, Andreas Roodt

**Affiliations:** a Department of Chemistry, University of the Free State, PO Box 339, Bloemfontein 9300, South Africa

## Abstract

In the title compound, [Hf(C_15_H_11_O_2_)_4_], the Hf^IV^ atom is coordinated by four 1,3-diphenyl­propane-1,3-dionato ligands with an average Hf—O distance of 2.17 (3) Å and O—Hf—O bite angles varying from 74.5 (1) to 75.02 (9)°. The coordination polyhedron shows a slightly distorted Archimedean square-anti­prismatic geometry. The crystal packing is stabilized by weak C—H⋯O inter­actions.

## Related literature

For a monoclinic isomorph of the title compound, see: Fay *et al.* (1979 ▶). For related literature on hafnium and zirconium diketonato complexes, see: Viljoen *et al.* (2008 ▶, 2009*a* ▶,*b* ▶, 2010 ▶); Steyn *et al.* (2008 ▶); Lewis & Fay (1974 ▶); Demakopoulos *et al.* (1995 ▶). For the use of acetyl­acetone in separation chemistry and homogenous catalysis, see: Van Aswegen *et al.* (1991 ▶); Steyn *et al.* (1992 ▶, 1997 ▶); Otto *et al.* (1998 ▶); Roodt & Steyn (2000 ▶); Brink *et al.* (2010 ▶). For a description of the Cambridge Structural Database, see: Allen (2002 ▶).
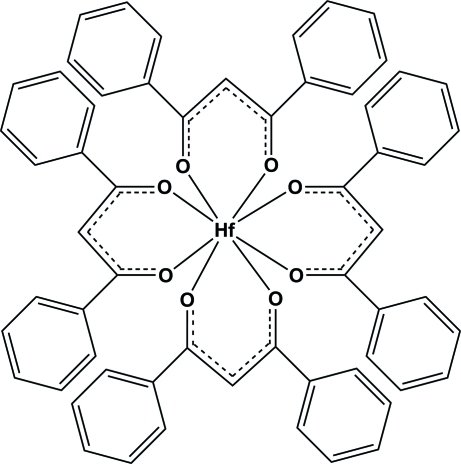


## Experimental

### 

#### Crystal data

[Hf(C_15_H_11_O_2_)_4_]
*M* *_r_* = 1071.44
Monoclinic, 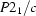

*a* = 24.846 (2) Å
*b* = 10.2236 (8) Å
*c* = 19.3155 (13) Å
β = 101.618 (4)°
*V* = 4805.8 (6) Å^3^
*Z* = 4
Mo *K*α radiation
μ = 2.23 mm^−1^
*T* = 100 K
0.20 × 0.19 × 0.11 mm

#### Data collection

Bruker X8 APEXII 4K KappaCCD diffractometer
Absorption correction: multi-scan (*SADABS*; Bruker, 2004 ▶) *T*_min_ = 0.661, *T*_max_ = 0.783
39626 measured reflections
11555 independent reflections
9232 reflections with *I* > 2σ(*I*)
*R*_int_ = 0.036

#### Refinement

*R*[*F* ^2^ > 2σ(*F* ^2^)] = 0.037
*wR*(*F* ^2^) = 0.122
*S* = 1.16
11555 reflections
623 parameters
H-atom parameters constrained
Δρ_max_ = 1.17 e Å^−3^
Δρ_min_ = −1.29 e Å^−3^

### 

Data collection: *APEX2* (Bruker, 2005 ▶); cell refinement: *SAINT-Plus* (Bruker, 2004 ▶); data reduction: *SAINT-Plus*; program(s) used to solve structure: *SIR92* (Altomare *et al.*, 1999 ▶); program(s) used to refine structure: *SHELXL97* (Sheldrick, 2008 ▶); molecular graphics: *DIAMOND* (Brandenburg & Putz, 2005 ▶); software used to prepare material for publication: *WinGX* (Farrugia, 1999 ▶).

## Supplementary Material

Crystal structure: contains datablocks I, global. DOI: 10.1107/S1600536810030400/bg2349sup1.cif

Structure factors: contains datablocks I. DOI: 10.1107/S1600536810030400/bg2349Isup2.hkl

Additional supplementary materials:  crystallographic information; 3D view; checkCIF report

## Figures and Tables

**Table 1:** Hydrogen-bond geometry (Å, °)

*D*—H⋯*A*	*D*—H	H⋯*A*	*D*⋯*A*	*D*—H⋯*A*
C43—H43⋯O6^i^	0.95	2.6	3.538 (5)	170

Symmetry code: (i) 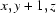.

**Fig. 1.**
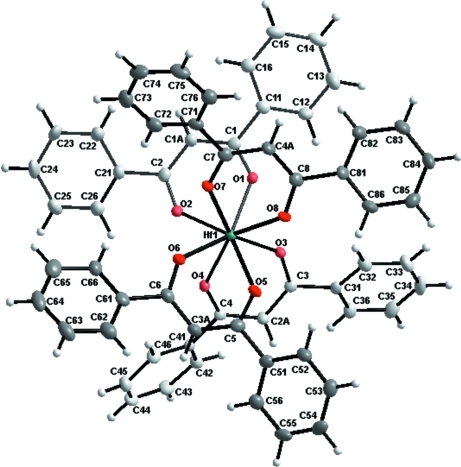
Representation of the title compound (I), showing the numbering scheme and displacement ellipsoids (50% probability).

**Fig. 2.**
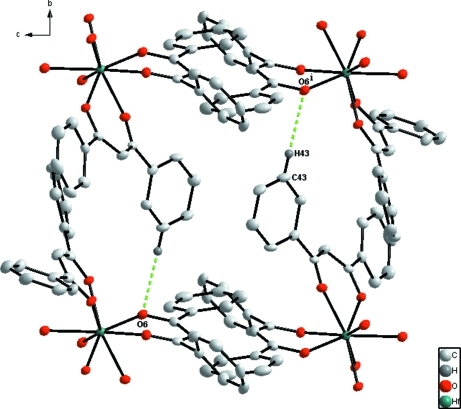
Graphical illustration of the title compound indicating the packing along the bc-plane (displacement ellipsoids at the 50% probability level. Non-interacting molecule portions and H atoms omitted for clarity). Symmetry code: (i) x, y + 1, z.

## supplementary crystallographic information

### Comment

Acetylacetone find applications as a ligand in the extraction and separation industry world-wide. However, it is also utilized in homogenous catalysis applications as a model precursor (Van Aswegen *et al.* (1991); Steyn *et al.* (1992, 1997); Otto *et al.* (1998); Roodt & Steyn, (2000); Brink *et al.* (2010)). This study forms part of ongoing research to investigate the reactions of *O*,*O*'- and *N*,*O*-bidentate ligands with hafnium(IV) and zirconium(IV) with possible applications in the mentioned industries (Steyn *et al.*, (2008); Viljoen *et al.* (2008, 2009*a*, 2009*b*, 2010); Demakopoulos *et al.* (1995) and Lewis & Fay (1974)).

Colourless cubic-like crystals of the title compound crystallize in the monoclinic crystal system (*P*2_1_*/c, Z*=4) (Figure 1). The Hf(IV) atom is eight coordinated and surrounded by four *β*-diketonate ligands, dibenzoylmethane (dbm^-^), adopting an Archimedean antiprismatic coordination geometry. The Hf—O bond lengths vary from 2.133 (2) Å to 2.200 (2) Å, with the average Hf—O distance being 2.169 (3) Å. This average Hf—O bond distance is somewhat larger than the average of 2.159 (5) Å obtained from the Cambridge Structural Database (Allen (2002)) (data extracted from 22 hits, yielding 60 observations ranging from 2.079 to 2.262 Å). The O—Hf—O bite angles vary between 74.5 (1) and 75.02 (9)°. The molecules of the title compound pack in horizontal layers along the *bc*-plane and are stabilized by weak C—H···O interactions (Table 1, Figure 2).

### Experimental

Chemicals were purchased from Sigma-Aldrich and used as received. HfCl_4_ (203 mg, 0.63 mmol) was dissolved in a minimal amount of DMF. While stirring this solution at room temperature, another solution of [C_15_H_12_O_2_] (568 mg, 2.5 mmol) was dissolved in a minimal amount of DMF and slowly added to the HfCl_4_ solution, resulting in the formation of a yellow solution, which was left to stand at 252 K for a few days after which colourless crystals, suitable for X-ray diffraction were obtained (Yield: 891 mg, 83%).

### Refinement

The aromatic, methine, and methyl H atoms were placed in geometrically idealized positions (C—H = 0.93–0.98) and constrained to ride on their parent atoms with *U*_iso_(H) = 1.2*U*_eq_(C) for aromatic and methine, and *U*_iso_(H) = 1.5*U*_eq_(C) for methyl protons. Torsion angles for methyl protons were refined from electron density. The highest residual electron density was located 0.7 Å from C314 and was essentially meaningless.

### Crystal data

**Table:**

[Hf(C_15_H_11_O_2_)_4_]	*F*(000) = 2160
*M**_r_* = 1071.44	*D*_x_ = 1.481 Mg m^−^^3^
Monoclinic, *P*2_1_/*c*	Mo *K*α radiation, λ = 0.71073 Å
Hall symbol: -P 2ybc	Cell parameters from 9989 reflections
*a* = 24.846 (2) Å	θ = 3.0–28.2°
*b* = 10.2236 (8) Å	µ = 2.23 mm^−^^1^
*c* = 19.3155 (13) Å	*T* = 100 K
β = 101.618 (4)°	Cuboid, colourless
*V* = 4805.8 (6) Å^3^	0.20 × 0.19 × 0.11 mm
*Z* = 4	

### Data collection

**Table:**

Bruker X8 APEXII 4K KappaCCD diffractometer	11555 independent reflections
Radiation source: fine-focus sealed tube	9232 reflections with *I* > 2σ(*I*)
graphite	*R*_int_ = 0.036
ω– and φ–scans	θ_max_ = 28°, θ_min_ = 0.8°
Absorption correction: multi-scan (*SADABS*; Bruker, 2004)	*h* = −23→32
*T*_min_ = 0.661, *T*_max_ = 0.783	*k* = −11→13
39626 measured reflections	*l* = −25→24

### Refinement

**Table:**

Refinement on *F*^2^	Secondary atom site location: difference Fourier map
Least-squares matrix: full	Hydrogen site location: inferred from neighbouring sites
*R*[*F*^2^ > 2σ(*F*^2^)] = 0.037	H-atom parameters constrained
*wR*(*F*^2^) = 0.122	*w* = 1/[σ^2^(*F*_o_^2^) + (0.0613*P*)^2^ + 3.4756*P*] where *P* = (*F*_o_^2^ + 2*F*_c_^2^)/3
*S* = 1.16	(Δ/σ)_max_ = 0.001
11555 reflections	Δρ_max_ = 1.17 e Å^−^^3^
623 parameters	Δρ_min_ = −1.29 e Å^−^^3^
0 restraints	Extinction correction: *SHELXL97* (Sheldrick, 2008)
Primary atom site location: structure-invariant direct methods	Extinction coefficient: 0.00501 (19)

### Special details

**Table:**

Geometry. All s.u.'s (except the s.u. in the dihedral angle between two l.s. planes) are estimated using the full covariance matrix. The cell s.u.'s are taken into account individually in the estimation of s.u.'s in distances, angles and torsion angles; correlations between s.u.'s in cell parameters are only used when they are defined by crystal symmetry. An approximate (isotropic) treatment of cell s.u.'s is used for estimating s.u.'s involving l.s. planes.
Refinement. Refinement of *F*^2^ against ALL reflections. The weighted *R*-factor *wR* and goodness of fit *S* are based on *F*^2^, conventional *R*-factors *R* are based on *F*, with *F* set to zero for negative *F*^2^. The threshold expression of *F*^2^ > 2σ(*F*^2^) is used only for calculating *R*-factors(gt) *etc*. and is not relevant to the choice of reflections for refinement. *R*-factors based on *F*^2^ are statistically about twice as large as those based on *F*, and *R*- factors based on ALL data will be even larger.

### Fractional atomic coordinates and isotropic or equivalent isotropic displacement parameters (Å^2^)

**Table:**

	*x*	*y*	*z*	*U*_iso_*/*U*_eq_	
C1	0.24706 (16)	0.4566 (4)	0.08242 (19)	0.0161 (8)	
C1A	0.30445 (16)	0.4590 (4)	0.0913 (2)	0.0207 (8)	
H1A	0.3209	0.4314	0.0534	0.025*	
C2	0.33800 (17)	0.5007 (3)	0.1542 (2)	0.0197 (8)	
C2A	0.20819 (15)	0.8200 (4)	0.29205 (19)	0.0187 (8)	
H2A	0.1954	0.8959	0.3121	0.022*	
C3	0.17177 (15)	0.7475 (3)	0.24237 (18)	0.0157 (7)	
C3A	0.28567 (17)	0.5107 (4)	0.4365 (2)	0.0208 (9)	
H3A	0.2987	0.5368	0.4841	0.025*	
C4	0.26320 (15)	0.7829 (4)	0.31278 (18)	0.0142 (7)	
C4A	0.19232 (16)	0.1980 (4)	0.1584 (2)	0.0228 (9)	
H4A	0.1779	0.133	0.1246	0.027*	
C5	0.23035 (18)	0.5291 (4)	0.4056 (2)	0.0197 (8)	
C6	0.32221 (16)	0.4545 (4)	0.3986 (2)	0.0201 (8)	
C7	0.24896 (16)	0.2030 (4)	0.18487 (19)	0.0201 (8)	
C8	0.15649 (16)	0.2868 (4)	0.1806 (2)	0.0213 (8)	
C11	0.21105 (16)	0.4190 (4)	0.01309 (19)	0.0189 (8)	
C12	0.15601 (17)	0.4562 (4)	−0.0013 (2)	0.0238 (9)	
H12	0.1417	0.5037	0.0333	0.029*	
C13	0.12182 (18)	0.4252 (4)	−0.0650 (2)	0.0301 (10)	
H13	0.0845	0.4526	−0.0746	0.036*	
C14	0.14269 (18)	0.3532 (4)	−0.1150 (2)	0.0268 (9)	
H14	0.1195	0.3318	−0.1589	0.032*	
C15	0.19669 (18)	0.3128 (4)	−0.1011 (2)	0.0283 (10)	
H15	0.2104	0.2625	−0.1351	0.034*	
C16	0.23119 (17)	0.3456 (4)	−0.0375 (2)	0.0243 (9)	
H16	0.2685	0.3182	−0.0283	0.029*	
C21	0.39823 (16)	0.5154 (4)	0.1617 (2)	0.0199 (8)	
C22	0.42742 (17)	0.4504 (5)	0.1174 (2)	0.0298 (10)	
H22	0.4086	0.3942	0.0813	0.036*	
C23	0.48370 (19)	0.4676 (5)	0.1259 (2)	0.0387 (12)	
H23	0.5034	0.4218	0.0961	0.046*	
C24	0.51146 (18)	0.5506 (6)	0.1772 (2)	0.0395 (12)	
H24	0.5499	0.5642	0.1819	0.047*	
C25	0.48275 (18)	0.6137 (5)	0.2215 (2)	0.0325 (10)	
H25	0.5017	0.6706	0.2572	0.039*	
C26	0.42671 (16)	0.5953 (4)	0.2148 (2)	0.0258 (9)	
H26	0.4077	0.6374	0.2467	0.031*	
C31	0.11385 (15)	0.7921 (4)	0.21507 (18)	0.0170 (8)	
C32	0.07438 (16)	0.6976 (4)	0.1882 (2)	0.0242 (9)	
H32	0.0845	0.6079	0.1893	0.029*	
C33	0.02068 (17)	0.7337 (4)	0.1600 (2)	0.0295 (10)	
H33	−0.0059	0.6687	0.1425	0.035*	
C34	0.00591 (17)	0.8638 (5)	0.1573 (2)	0.0290 (10)	
H34	−0.0308	0.8883	0.1373	0.035*	
C35	0.04449 (18)	0.9597 (4)	0.1835 (2)	0.0260 (9)	
H35	0.0343	1.0494	0.1812	0.031*	
C36	0.09820 (16)	0.9229 (4)	0.21317 (19)	0.0208 (8)	
H36	0.1244	0.9878	0.2323	0.025*	
C41	0.30414 (15)	0.8754 (4)	0.35389 (18)	0.0166 (8)	
C42	0.29894 (17)	1.0095 (4)	0.3414 (2)	0.0197 (8)	
H42	0.2678	1.0425	0.3093	0.024*	
C43	0.33883 (17)	1.0947 (4)	0.3756 (2)	0.0241 (9)	
H43	0.3361	1.1854	0.3649	0.029*	
C44	0.38253 (16)	1.0485 (4)	0.4251 (2)	0.0228 (9)	
H44	0.4095	1.1074	0.4492	0.027*	
C45	0.38700 (16)	0.9157 (4)	0.4397 (2)	0.0242 (9)	
H45	0.4163	0.884	0.475	0.029*	
C46	0.34863 (16)	0.8288 (4)	0.4026 (2)	0.0211 (8)	
H46	0.353	0.7374	0.4108	0.025*	
C51	0.19077 (16)	0.5727 (4)	0.44986 (19)	0.0209 (8)	
C52	0.13972 (18)	0.5118 (4)	0.4416 (2)	0.0240 (9)	
H52	0.1295	0.447	0.4062	0.029*	
C53	0.10394 (18)	0.5455 (4)	0.4850 (2)	0.0276 (9)	
H53	0.0696	0.502	0.4803	0.033*	
C54	0.11806 (17)	0.6423 (4)	0.5350 (2)	0.0275 (10)	
H54	0.0933	0.6651	0.5646	0.033*	
C55	0.16760 (18)	0.7061 (4)	0.5423 (2)	0.0287 (10)	
H55	0.1766	0.7737	0.5764	0.034*	
C56	0.20449 (17)	0.6720 (4)	0.5000 (2)	0.0261 (9)	
H56	0.2388	0.7158	0.5052	0.031*	
C61	0.37973 (16)	0.4165 (4)	0.4335 (2)	0.0216 (8)	
C62	0.39500 (18)	0.3968 (4)	0.5053 (2)	0.0307 (10)	
H62	0.3693	0.4094	0.5349	0.037*	
C63	0.44867 (19)	0.3583 (4)	0.5339 (2)	0.0345 (11)	
H63	0.459	0.3441	0.5834	0.041*	
C64	0.48704 (19)	0.3402 (4)	0.4925 (2)	0.0345 (11)	
H64	0.5236	0.3151	0.513	0.041*	
C65	0.47105 (19)	0.3596 (4)	0.4190 (3)	0.0337 (10)	
H65	0.4967	0.3467	0.3891	0.04*	
C66	0.41714 (16)	0.3980 (4)	0.3904 (2)	0.0251 (9)	
H66	0.4062	0.4115	0.3409	0.03*	
C71	0.28609 (17)	0.0940 (4)	0.17170 (19)	0.0218 (8)	
C72	0.34175 (18)	0.1171 (4)	0.1818 (2)	0.0270 (9)	
H72	0.3556	0.2022	0.1946	0.032*	
C73	0.3775 (2)	0.0184 (5)	0.1735 (2)	0.0328 (11)	
H73	0.4157	0.036	0.1796	0.039*	
C74	0.3580 (2)	−0.1065 (5)	0.1563 (2)	0.0337 (11)	
H74	0.3828	−0.1747	0.1507	0.04*	
C75	0.3029 (2)	−0.1320 (4)	0.1475 (2)	0.0313 (10)	
H75	0.2896	−0.2182	0.1365	0.038*	
C76	0.26652 (19)	−0.0328 (4)	0.1544 (2)	0.0286 (10)	
H76	0.2283	−0.0506	0.1475	0.034*	
C81	0.09616 (16)	0.2845 (4)	0.1502 (2)	0.0203 (8)	
C82	0.07572 (17)	0.2409 (4)	0.0814 (2)	0.0258 (9)	
H82	0.1003	0.2083	0.0536	0.031*	
C83	0.02023 (18)	0.2449 (4)	0.0537 (2)	0.0293 (10)	
H83	0.0066	0.2158	0.0067	0.035*	
C84	−0.01538 (17)	0.2907 (4)	0.0934 (2)	0.0273 (9)	
H84	−0.0536	0.2934	0.0738	0.033*	
C85	0.00363 (17)	0.3330 (4)	0.1619 (2)	0.0266 (9)	
H85	−0.0215	0.3628	0.1895	0.032*	
C86	0.05943 (17)	0.3319 (4)	0.1901 (2)	0.0239 (9)	
H86	0.0727	0.3634	0.2367	0.029*	
O1	0.22174 (11)	0.4883 (2)	0.13129 (13)	0.0159 (6)	
O3	0.18452 (10)	0.6395 (2)	0.21599 (12)	0.0145 (5)	
O4	0.28238 (10)	0.6747 (2)	0.29717 (12)	0.0154 (5)	
O5	0.20986 (12)	0.5050 (2)	0.34095 (14)	0.0179 (6)	
O6	0.30933 (10)	0.4271 (3)	0.33300 (12)	0.0181 (5)	
O7	0.27280 (10)	0.2980 (2)	0.22244 (12)	0.0164 (5)	
O8	0.17248 (10)	0.3757 (3)	0.22776 (12)	0.0177 (6)	
O2	0.31948 (11)	0.5335 (3)	0.20904 (13)	0.0173 (5)	
Hf1	0.245894 (6)	0.493352 (14)	0.247129 (7)	0.01434 (8)	

### Atomic displacement parameters (Å^2^)

**Table:**

	*U*^11^	*U*^22^	*U*^33^	*U*^12^	*U*^13^	*U*^23^
C1	0.021 (2)	0.0103 (18)	0.0170 (17)	0.0021 (15)	0.0036 (15)	0.0021 (15)
C1A	0.018 (2)	0.025 (2)	0.0190 (18)	0.0002 (17)	0.0037 (15)	−0.0020 (17)
C2	0.018 (2)	0.016 (2)	0.024 (2)	0.0030 (15)	0.0039 (16)	0.0017 (15)
C2A	0.0167 (19)	0.015 (2)	0.0239 (19)	0.0005 (15)	0.0025 (15)	−0.0046 (15)
C3	0.0178 (19)	0.0123 (18)	0.0174 (17)	−0.0002 (14)	0.0048 (14)	0.0032 (14)
C3A	0.022 (2)	0.022 (2)	0.0170 (18)	−0.0039 (16)	0.0015 (15)	−0.0018 (15)
C4	0.0164 (18)	0.0108 (18)	0.0163 (16)	−0.0025 (14)	0.0055 (14)	0.0010 (14)
C4A	0.021 (2)	0.021 (2)	0.026 (2)	−0.0054 (16)	0.0040 (16)	−0.0065 (17)
C5	0.026 (2)	0.0120 (19)	0.0212 (19)	−0.0039 (16)	0.0063 (16)	0.0005 (15)
C6	0.021 (2)	0.016 (2)	0.0208 (18)	−0.0045 (16)	−0.0005 (15)	0.0021 (16)
C7	0.025 (2)	0.015 (2)	0.0204 (18)	−0.0040 (16)	0.0061 (16)	0.0031 (15)
C8	0.024 (2)	0.018 (2)	0.0233 (19)	−0.0061 (16)	0.0075 (16)	−0.0003 (16)
C11	0.022 (2)	0.0142 (19)	0.0198 (18)	−0.0030 (15)	0.0027 (15)	0.0016 (15)
C12	0.025 (2)	0.023 (2)	0.024 (2)	−0.0009 (17)	0.0044 (16)	−0.0068 (17)
C13	0.025 (2)	0.026 (2)	0.035 (2)	0.0007 (18)	−0.0059 (18)	0.0003 (19)
C14	0.038 (3)	0.022 (2)	0.0174 (18)	−0.0104 (19)	−0.0014 (17)	−0.0024 (16)
C15	0.039 (3)	0.025 (2)	0.022 (2)	−0.0079 (19)	0.0099 (18)	−0.0046 (17)
C16	0.026 (2)	0.023 (2)	0.025 (2)	−0.0014 (17)	0.0052 (16)	−0.0019 (17)
C21	0.0156 (19)	0.022 (2)	0.0218 (19)	0.0000 (15)	0.0036 (15)	0.0013 (15)
C22	0.021 (2)	0.043 (3)	0.025 (2)	0.003 (2)	0.0041 (17)	−0.007 (2)
C23	0.020 (2)	0.067 (3)	0.031 (2)	0.006 (2)	0.0116 (19)	−0.012 (2)
C24	0.014 (2)	0.073 (4)	0.032 (2)	−0.006 (2)	0.0043 (18)	−0.003 (2)
C25	0.025 (2)	0.045 (3)	0.027 (2)	−0.008 (2)	0.0033 (17)	−0.006 (2)
C26	0.021 (2)	0.032 (2)	0.0240 (19)	0.0001 (18)	0.0034 (16)	−0.0010 (18)
C31	0.0157 (19)	0.022 (2)	0.0140 (16)	0.0016 (15)	0.0036 (14)	0.0027 (15)
C32	0.022 (2)	0.025 (2)	0.025 (2)	−0.0020 (17)	0.0014 (16)	−0.0002 (17)
C33	0.021 (2)	0.033 (3)	0.031 (2)	−0.0063 (18)	−0.0033 (17)	−0.0020 (19)
C34	0.015 (2)	0.043 (3)	0.028 (2)	0.0058 (19)	0.0021 (16)	0.012 (2)
C35	0.026 (2)	0.024 (2)	0.030 (2)	0.0072 (18)	0.0090 (18)	0.0088 (18)
C36	0.0182 (19)	0.022 (2)	0.0228 (18)	−0.0008 (16)	0.0057 (15)	0.0041 (16)
C41	0.0126 (18)	0.019 (2)	0.0191 (17)	−0.0039 (15)	0.0055 (14)	−0.0040 (15)
C42	0.020 (2)	0.017 (2)	0.0212 (19)	−0.0006 (15)	0.0015 (15)	0.0007 (15)
C43	0.027 (2)	0.014 (2)	0.032 (2)	−0.0042 (17)	0.0066 (17)	−0.0036 (17)
C44	0.020 (2)	0.020 (2)	0.029 (2)	−0.0085 (17)	0.0050 (16)	−0.0074 (17)
C45	0.018 (2)	0.024 (2)	0.029 (2)	−0.0011 (17)	0.0001 (16)	−0.0006 (17)
C46	0.022 (2)	0.0108 (19)	0.030 (2)	−0.0027 (15)	0.0049 (16)	−0.0007 (16)
C51	0.026 (2)	0.021 (2)	0.0166 (17)	0.0013 (17)	0.0044 (15)	0.0007 (16)
C52	0.026 (2)	0.019 (2)	0.025 (2)	0.0007 (16)	−0.0006 (17)	−0.0030 (16)
C53	0.023 (2)	0.027 (2)	0.033 (2)	0.0009 (18)	0.0058 (18)	0.0056 (19)
C54	0.030 (2)	0.037 (3)	0.0166 (18)	0.0130 (19)	0.0052 (16)	0.0044 (18)
C55	0.037 (3)	0.027 (2)	0.0186 (19)	0.0100 (19)	−0.0034 (17)	−0.0061 (17)
C56	0.025 (2)	0.029 (2)	0.0233 (19)	−0.0026 (18)	0.0027 (16)	0.0008 (17)
C61	0.023 (2)	0.014 (2)	0.0264 (19)	−0.0051 (16)	0.0013 (16)	−0.0039 (16)
C62	0.033 (3)	0.024 (2)	0.032 (2)	−0.0009 (19)	−0.0030 (18)	−0.0005 (18)
C63	0.032 (3)	0.027 (3)	0.037 (2)	−0.005 (2)	−0.010 (2)	0.001 (2)
C64	0.023 (2)	0.018 (2)	0.057 (3)	−0.0004 (18)	−0.007 (2)	−0.003 (2)
C65	0.030 (2)	0.019 (2)	0.053 (3)	−0.0052 (18)	0.010 (2)	−0.005 (2)
C66	0.020 (2)	0.022 (2)	0.031 (2)	−0.0027 (17)	0.0019 (17)	−0.0037 (17)
C71	0.028 (2)	0.020 (2)	0.0167 (18)	0.0017 (17)	0.0022 (15)	−0.0021 (16)
C72	0.033 (2)	0.024 (2)	0.022 (2)	0.0001 (18)	−0.0002 (17)	−0.0012 (17)
C73	0.025 (2)	0.037 (3)	0.032 (2)	0.0027 (19)	−0.0020 (19)	0.001 (2)
C74	0.041 (3)	0.028 (3)	0.029 (2)	0.015 (2)	0.002 (2)	−0.0039 (19)
C75	0.046 (3)	0.017 (2)	0.027 (2)	−0.001 (2)	−0.0010 (19)	−0.0027 (18)
C76	0.030 (2)	0.028 (2)	0.026 (2)	−0.0009 (19)	0.0043 (18)	−0.0005 (18)
C81	0.017 (2)	0.016 (2)	0.0268 (19)	−0.0059 (15)	0.0041 (15)	−0.0020 (16)
C82	0.025 (2)	0.028 (2)	0.025 (2)	−0.0074 (18)	0.0082 (17)	−0.0073 (17)
C83	0.029 (2)	0.028 (2)	0.028 (2)	−0.0022 (19)	−0.0019 (18)	−0.0092 (18)
C84	0.019 (2)	0.025 (2)	0.033 (2)	0.0022 (17)	−0.0048 (17)	−0.0047 (18)
C85	0.026 (2)	0.020 (2)	0.036 (2)	0.0004 (17)	0.0123 (18)	−0.0032 (18)
C86	0.028 (2)	0.023 (2)	0.0186 (18)	−0.0041 (17)	0.0012 (16)	−0.0050 (16)
O1	0.0136 (13)	0.0162 (14)	0.0177 (13)	0.0015 (10)	0.0029 (10)	−0.0022 (10)
O3	0.0143 (13)	0.0129 (13)	0.0153 (12)	0.0005 (10)	0.0002 (10)	−0.0022 (10)
O4	0.0126 (13)	0.0136 (13)	0.0191 (12)	0.0007 (10)	0.0014 (10)	−0.0004 (10)
O5	0.0192 (14)	0.0181 (15)	0.0147 (12)	−0.0031 (10)	−0.0004 (11)	−0.0013 (10)
O6	0.0188 (14)	0.0159 (14)	0.0183 (12)	0.0020 (11)	0.0003 (10)	−0.0039 (11)
O7	0.0167 (13)	0.0135 (13)	0.0180 (12)	−0.0011 (10)	0.0014 (10)	−0.0041 (10)
O8	0.0192 (14)	0.0171 (14)	0.0169 (12)	−0.0052 (11)	0.0038 (10)	−0.0058 (10)
O2	0.0147 (13)	0.0177 (14)	0.0196 (13)	−0.0003 (11)	0.0036 (10)	−0.0051 (11)
Hf1	0.01395 (10)	0.01391 (11)	0.01428 (10)	−0.00034 (6)	0.00073 (6)	−0.00185 (6)

### Geometric parameters (Å, °)

**Table:**

C1—O1	1.277 (4)	C41—C42	1.393 (5)
C1—C1A	1.402 (5)	C42—C43	1.383 (5)
C1—C11	1.503 (5)	C42—H42	0.95
C1A—C2	1.396 (5)	C43—C44	1.378 (6)
C1A—H1A	0.95	C43—H43	0.95
C2—O2	1.281 (5)	C44—C45	1.386 (6)
C2—C21	1.482 (6)	C44—H44	0.95
C2A—C3	1.393 (5)	C45—C46	1.391 (5)
C2A—C4	1.397 (5)	C45—H45	0.95
C2A—H2A	0.95	C46—H46	0.95
C3—O3	1.282 (4)	C51—C52	1.393 (6)
C3—C31	1.501 (5)	C51—C56	1.397 (5)
C3A—C5	1.397 (6)	C52—C53	1.382 (6)
C3A—C6	1.399 (6)	C52—H52	0.95
C3A—H3A	0.95	C53—C54	1.378 (6)
C4—O4	1.264 (4)	C53—H53	0.95
C4—C41	1.495 (5)	C54—C55	1.375 (6)
C4A—C7	1.398 (5)	C54—H54	0.95
C4A—C8	1.399 (5)	C55—C56	1.389 (6)
C4A—H4A	0.95	C55—H55	0.95
C5—O5	1.273 (5)	C56—H56	0.95
C5—C51	1.496 (5)	C61—C62	1.376 (5)
C6—O6	1.274 (4)	C61—C66	1.379 (5)
C6—C61	1.503 (5)	C62—C63	1.393 (6)
C7—O7	1.284 (4)	C62—H62	0.95
C7—C71	1.500 (6)	C63—C64	1.375 (6)
C8—O8	1.291 (4)	C63—H63	0.95
C8—C81	1.496 (5)	C64—C65	1.408 (6)
C11—C12	1.392 (5)	C64—H64	0.95
C11—C16	1.402 (5)	C65—C66	1.398 (6)
C12—C13	1.385 (5)	C65—H65	0.95
C12—H12	0.95	C66—H66	0.95
C13—C14	1.394 (6)	C71—C72	1.378 (6)
C13—H13	0.95	C71—C76	1.401 (6)
C14—C15	1.377 (6)	C72—C73	1.375 (6)
C14—H14	0.95	C72—H72	0.95
C15—C16	1.389 (5)	C73—C74	1.382 (6)
C15—H15	0.95	C73—H73	0.95
C16—H16	0.95	C74—C75	1.370 (6)
C21—C26	1.389 (5)	C74—H74	0.95
C21—C22	1.397 (6)	C75—C76	1.382 (6)
C22—C23	1.386 (6)	C75—H75	0.95
C22—H22	0.95	C76—H76	0.95
C23—C24	1.380 (7)	C81—C86	1.395 (5)
C23—H23	0.95	C81—C82	1.397 (5)
C24—C25	1.380 (6)	C82—C83	1.375 (6)
C24—H24	0.95	C82—H82	0.95
C25—C26	1.385 (6)	C83—C84	1.365 (6)
C25—H25	0.95	C83—H83	0.95
C26—H26	0.95	C84—C85	1.381 (6)
C31—C36	1.391 (5)	C84—H84	0.95
C31—C32	1.400 (5)	C85—C86	1.384 (6)
C32—C33	1.386 (6)	C85—H85	0.95
C32—H32	0.95	C86—H86	0.95
C33—C34	1.379 (6)	O1—Hf1	2.197 (3)
C33—H33	0.95	O3—Hf1	2.133 (2)
C34—C35	1.393 (6)	O4—Hf1	2.200 (2)
C34—H34	0.95	O5—Hf1	2.180 (3)
C35—C36	1.394 (5)	O6—Hf1	2.154 (2)
C35—H35	0.95	O7—Hf1	2.189 (2)
C36—H36	0.95	O8—Hf1	2.154 (2)
C41—C46	1.384 (5)	O2—Hf1	2.143 (3)
			
O1—C1—C1A	123.1 (3)	C52—C51—C56	119.5 (4)
O1—C1—C11	115.5 (3)	C52—C51—C5	119.3 (4)
C1A—C1—C11	121.5 (3)	C56—C51—C5	121.1 (4)
C2—C1A—C1	121.8 (4)	C53—C52—C51	120.1 (4)
C2—C1A—H1A	119.1	C53—C52—H52	119.9
C1—C1A—H1A	119.1	C51—C52—H52	119.9
O2—C2—C1A	123.3 (4)	C54—C53—C52	120.0 (4)
O2—C2—C21	114.6 (4)	C54—C53—H53	120
C1A—C2—C21	122.0 (4)	C52—C53—H53	120
C3—C2A—C4	121.0 (3)	C55—C54—C53	120.6 (4)
C3—C2A—H2A	119.5	C55—C54—H54	119.7
C4—C2A—H2A	119.5	C53—C54—H54	119.7
O3—C3—C2A	123.7 (3)	C54—C55—C56	120.2 (4)
O3—C3—C31	114.7 (3)	C54—C55—H55	119.9
C2A—C3—C31	121.6 (3)	C56—C55—H55	119.9
C5—C3A—C6	121.2 (4)	C55—C56—C51	119.5 (4)
C5—C3A—H3A	119.4	C55—C56—H56	120.2
C6—C3A—H3A	119.4	C51—C56—H56	120.2
O4—C4—C2A	124.3 (3)	C62—C61—C66	120.3 (4)
O4—C4—C41	115.6 (3)	C62—C61—C6	122.3 (4)
C2A—C4—C41	120.1 (3)	C66—C61—C6	117.4 (3)
C7—C4A—C8	121.3 (4)	C61—C62—C63	119.2 (4)
C7—C4A—H4A	119.3	C61—C62—H62	120.4
C8—C4A—H4A	119.3	C63—C62—H62	120.4
O5—C5—C3A	123.9 (4)	C64—C63—C62	121.8 (4)
O5—C5—C51	116.1 (4)	C64—C63—H63	119.1
C3A—C5—C51	119.9 (4)	C62—C63—H63	119.1
O6—C6—C3A	123.4 (4)	C63—C64—C65	118.6 (4)
O6—C6—C61	114.5 (3)	C63—C64—H64	120.7
C3A—C6—C61	122.0 (3)	C65—C64—H64	120.7
O7—C7—C4A	123.4 (4)	C66—C65—C64	119.4 (4)
O7—C7—C71	115.4 (3)	C66—C65—H65	120.3
C4A—C7—C71	121.3 (4)	C64—C65—H65	120.3
O8—C8—C4A	123.3 (4)	C61—C66—C65	120.6 (4)
O8—C8—C81	115.5 (3)	C61—C66—H66	119.7
C4A—C8—C81	121.2 (3)	C65—C66—H66	119.7
C12—C11—C16	118.8 (3)	C72—C71—C76	118.8 (4)
C12—C11—C1	119.5 (3)	C72—C71—C7	118.9 (4)
C16—C11—C1	121.7 (3)	C76—C71—C7	122.1 (4)
C13—C12—C11	121.1 (4)	C73—C72—C71	120.8 (4)
C13—C12—H12	119.5	C73—C72—H72	119.6
C11—C12—H12	119.5	C71—C72—H72	119.6
C12—C13—C14	119.3 (4)	C72—C73—C74	120.2 (5)
C12—C13—H13	120.3	C72—C73—H73	119.9
C14—C13—H13	120.3	C74—C73—H73	119.9
C15—C14—C13	120.4 (4)	C75—C74—C73	119.9 (4)
C15—C14—H14	119.8	C75—C74—H74	120
C13—C14—H14	119.8	C73—C74—H74	120
C14—C15—C16	120.3 (4)	C74—C75—C76	120.3 (4)
C14—C15—H15	119.9	C74—C75—H75	119.8
C16—C15—H15	119.9	C76—C75—H75	119.8
C15—C16—C11	120.1 (4)	C75—C76—C71	120.0 (4)
C15—C16—H16	120	C75—C76—H76	120
C11—C16—H16	120	C71—C76—H76	120
C26—C21—C22	118.9 (4)	C86—C81—C82	118.9 (4)
C26—C21—C2	119.0 (4)	C86—C81—C8	119.5 (3)
C22—C21—C2	122.1 (4)	C82—C81—C8	121.5 (4)
C23—C22—C21	120.2 (4)	C83—C82—C81	120.3 (4)
C23—C22—H22	119.9	C83—C82—H82	119.9
C21—C22—H22	119.9	C81—C82—H82	119.9
C24—C23—C22	120.6 (4)	C84—C83—C82	120.3 (4)
C24—C23—H23	119.7	C84—C83—H83	119.9
C22—C23—H23	119.7	C82—C83—H83	119.9
C23—C24—C25	119.2 (4)	C83—C84—C85	120.7 (4)
C23—C24—H24	120.4	C83—C84—H84	119.7
C25—C24—H24	120.4	C85—C84—H84	119.7
C24—C25—C26	120.9 (4)	C84—C85—C86	119.8 (4)
C24—C25—H25	119.5	C84—C85—H85	120.1
C26—C25—H25	119.5	C86—C85—H85	120.1
C25—C26—C21	120.1 (4)	C85—C86—C81	120.0 (4)
C25—C26—H26	119.9	C85—C86—H86	120
C21—C26—H26	119.9	C81—C86—H86	120
C36—C31—C32	118.9 (4)	C1—O1—Hf1	133.2 (2)
C36—C31—C3	123.0 (3)	C3—O3—Hf1	135.3 (2)
C32—C31—C3	118.1 (3)	C4—O4—Hf1	134.4 (2)
C33—C32—C31	120.6 (4)	C5—O5—Hf1	132.5 (3)
C33—C32—H32	119.7	C6—O6—Hf1	134.0 (2)
C31—C32—H32	119.7	C7—O7—Hf1	133.4 (2)
C34—C33—C32	120.0 (4)	C8—O8—Hf1	130.2 (2)
C34—C33—H33	120	C2—O2—Hf1	135.0 (2)
C32—C33—H33	120	O3—Hf1—O2	112.32 (10)
C33—C34—C35	120.5 (4)	O3—Hf1—O8	79.05 (10)
C33—C34—H34	119.8	O2—Hf1—O8	142.98 (9)
C35—C34—H34	119.8	O3—Hf1—O6	143.90 (9)
C34—C35—C36	119.5 (4)	O2—Hf1—O6	77.42 (10)
C34—C35—H35	120.3	O8—Hf1—O6	114.87 (10)
C36—C35—H35	120.3	O3—Hf1—O5	78.83 (9)
C31—C36—C35	120.6 (4)	O2—Hf1—O5	142.78 (10)
C31—C36—H36	119.7	O8—Hf1—O5	72.53 (9)
C35—C36—H36	119.7	O6—Hf1—O5	74.71 (10)
C46—C41—C42	119.3 (4)	O3—Hf1—O7	144.09 (9)
C46—C41—C4	120.5 (3)	O2—Hf1—O7	77.35 (10)
C42—C41—C4	120.1 (3)	O8—Hf1—O7	74.79 (9)
C43—C42—C41	120.3 (4)	O6—Hf1—O7	70.89 (9)
C43—C42—H42	119.9	O5—Hf1—O7	115.18 (9)
C41—C42—H42	119.9	O3—Hf1—O1	72.31 (9)
C44—C43—C42	120.3 (4)	O2—Hf1—O1	74.45 (10)
C44—C43—H43	119.9	O8—Hf1—O1	76.23 (9)
C42—C43—H43	119.9	O6—Hf1—O1	141.63 (9)
C43—C44—C45	119.8 (4)	O5—Hf1—O1	140.72 (10)
C43—C44—H44	120.1	O7—Hf1—O1	77.85 (9)
C45—C44—H44	120.1	O3—Hf1—O4	75.02 (9)
C44—C45—C46	120.2 (4)	O2—Hf1—O4	71.63 (9)
C44—C45—H45	119.9	O8—Hf1—O4	143.76 (9)
C46—C45—H45	119.9	O6—Hf1—O4	75.76 (9)
C41—C46—C45	120.0 (4)	O5—Hf1—O4	77.92 (9)
C41—C46—H46	120	O7—Hf1—O4	138.39 (9)
C45—C46—H46	120	O1—Hf1—O4	118.10 (9)
			
O1—C1—C1A—C2	−2.1 (6)	C76—C71—C72—C73	1.5 (6)
C11—C1—C1A—C2	176.4 (4)	C7—C71—C72—C73	177.0 (4)
C1—C1A—C2—O2	3.5 (6)	C71—C72—C73—C74	−1.4 (6)
C1—C1A—C2—C21	−174.1 (4)	C72—C73—C74—C75	0.1 (7)
C4—C2A—C3—O3	4.6 (6)	C73—C74—C75—C76	1.1 (7)
C4—C2A—C3—C31	−174.8 (3)	C74—C75—C76—C71	−1.0 (6)
C3—C2A—C4—O4	−10.5 (6)	C72—C71—C76—C75	−0.3 (6)
C3—C2A—C4—C41	167.1 (3)	C7—C71—C76—C75	−175.6 (4)
C6—C3A—C5—O5	−4.7 (6)	O8—C8—C81—C86	−26.4 (5)
C6—C3A—C5—C51	171.9 (4)	C4A—C8—C81—C86	153.8 (4)
C5—C3A—C6—O6	6.2 (6)	O8—C8—C81—C82	150.9 (4)
C5—C3A—C6—C61	−171.2 (4)	C4A—C8—C81—C82	−28.9 (6)
C8—C4A—C7—O7	−10.5 (6)	C86—C81—C82—C83	0.1 (6)
C8—C4A—C7—C71	168.5 (4)	C8—C81—C82—C83	−177.2 (4)
C7—C4A—C8—O8	−2.1 (6)	C81—C82—C83—C84	−0.5 (7)
C7—C4A—C8—C81	177.7 (4)	C82—C83—C84—C85	−0.3 (7)
O1—C1—C11—C12	18.8 (5)	C83—C84—C85—C86	1.4 (7)
C1A—C1—C11—C12	−159.8 (4)	C84—C85—C86—C81	−1.9 (6)
O1—C1—C11—C16	−160.3 (4)	C82—C81—C86—C85	1.1 (6)
C1A—C1—C11—C16	21.1 (6)	C8—C81—C86—C85	178.5 (4)
C16—C11—C12—C13	−1.9 (6)	C1A—C1—O1—Hf1	−23.4 (6)
C1—C11—C12—C13	178.9 (4)	C11—C1—O1—Hf1	158.0 (2)
C11—C12—C13—C14	1.3 (7)	C2A—C3—O3—Hf1	21.8 (5)
C12—C13—C14—C15	0.3 (6)	C31—C3—O3—Hf1	−158.7 (2)
C13—C14—C15—C16	−1.2 (6)	C2A—C4—O4—Hf1	−8.3 (5)
C14—C15—C16—C11	0.5 (6)	C41—C4—O4—Hf1	174.0 (2)
C12—C11—C16—C15	1.0 (6)	C3A—C5—O5—Hf1	−23.4 (6)
C1—C11—C16—C15	−179.9 (4)	C51—C5—O5—Hf1	159.9 (2)
O2—C2—C21—C26	−18.7 (5)	C3A—C6—O6—Hf1	20.9 (6)
C1A—C2—C21—C26	159.1 (4)	C61—C6—O6—Hf1	−161.4 (2)
O2—C2—C21—C22	160.7 (4)	C4A—C7—O7—Hf1	−11.6 (5)
C1A—C2—C21—C22	−21.5 (6)	C71—C7—O7—Hf1	169.4 (2)
C26—C21—C22—C23	−1.1 (7)	C4A—C8—O8—Hf1	37.5 (5)
C2—C21—C22—C23	179.5 (4)	C81—C8—O8—Hf1	−142.4 (3)
C21—C22—C23—C24	−1.1 (8)	C1A—C2—O2—Hf1	21.9 (6)
C22—C23—C24—C25	1.9 (8)	C21—C2—O2—Hf1	−160.4 (3)
C23—C24—C25—C26	−0.4 (8)	C3—O3—Hf1—O2	−89.8 (3)
C24—C25—C26—C21	−1.9 (7)	C3—O3—Hf1—O8	127.1 (3)
C22—C21—C26—C25	2.6 (6)	C3—O3—Hf1—O6	9.6 (4)
C2—C21—C26—C25	−178.0 (4)	C3—O3—Hf1—O5	53.0 (3)
O3—C3—C31—C36	−153.1 (3)	C3—O3—Hf1—O7	170.8 (3)
C2A—C3—C31—C36	26.3 (5)	C3—O3—Hf1—O1	−154.0 (3)
O3—C3—C31—C32	24.7 (5)	C3—O3—Hf1—O4	−27.3 (3)
C2A—C3—C31—C32	−155.9 (3)	C2—O2—Hf1—O3	−93.3 (3)
C36—C31—C32—C33	0.2 (6)	C2—O2—Hf1—O8	8.5 (4)
C3—C31—C32—C33	−177.7 (3)	C2—O2—Hf1—O6	123.3 (3)
C31—C32—C33—C34	0.9 (6)	C2—O2—Hf1—O5	165.5 (3)
C32—C33—C34—C35	−0.8 (6)	C2—O2—Hf1—O7	50.4 (3)
C33—C34—C35—C36	−0.5 (6)	C2—O2—Hf1—O1	−30.3 (3)
C32—C31—C36—C35	−1.5 (5)	C2—O2—Hf1—O4	−157.8 (4)
C3—C31—C36—C35	176.3 (3)	C8—O8—Hf1—O3	114.8 (3)
C34—C35—C36—C31	1.7 (6)	C8—O8—Hf1—O2	2.1 (4)
O4—C4—C41—C46	−33.8 (5)	C8—O8—Hf1—O6	−100.3 (3)
C2A—C4—C41—C46	148.4 (4)	C8—O8—Hf1—O5	−163.6 (3)
O4—C4—C41—C42	143.7 (4)	C8—O8—Hf1—O7	−40.3 (3)
C2A—C4—C41—C42	−34.1 (5)	C8—O8—Hf1—O1	40.6 (3)
C46—C41—C42—C43	2.2 (6)	C8—O8—Hf1—O4	159.6 (3)
C4—C41—C42—C43	−175.3 (3)	C6—O6—Hf1—O3	13.0 (4)
C41—C42—C43—C44	−3.6 (6)	C6—O6—Hf1—O2	123.7 (4)
C42—C43—C44—C45	1.3 (6)	C6—O6—Hf1—O8	−93.3 (3)
C43—C44—C45—C46	2.3 (6)	C6—O6—Hf1—O5	−31.3 (3)
C42—C41—C46—C45	1.3 (6)	C6—O6—Hf1—O7	−155.5 (4)
C4—C41—C46—C45	178.8 (3)	C6—O6—Hf1—O1	167.3 (3)
C44—C45—C46—C41	−3.6 (6)	C6—O6—Hf1—O4	49.8 (3)
O5—C5—C51—C52	41.3 (5)	C5—O5—Hf1—O3	−123.0 (3)
C3A—C5—C51—C52	−135.5 (4)	C5—O5—Hf1—O2	−10.6 (4)
O5—C5—C51—C56	−139.7 (4)	C5—O5—Hf1—O8	155.1 (3)
C3A—C5—C51—C56	43.4 (6)	C5—O5—Hf1—O6	32.2 (3)
C56—C51—C52—C53	−2.8 (6)	C5—O5—Hf1—O7	92.0 (3)
C5—C51—C52—C53	176.2 (4)	C5—O5—Hf1—O1	−166.0 (3)
C51—C52—C53—C54	2.0 (6)	C5—O5—Hf1—O4	−46.1 (3)
C52—C53—C54—C55	0.0 (6)	C7—O7—Hf1—O3	−16.6 (4)
C53—C54—C55—C56	−1.1 (6)	C7—O7—Hf1—O2	−127.3 (3)
C54—C55—C56—C51	0.3 (6)	C7—O7—Hf1—O8	28.0 (3)
C52—C51—C56—C55	1.7 (6)	C7—O7—Hf1—O6	151.8 (3)
C5—C51—C56—C55	−177.3 (4)	C7—O7—Hf1—O5	89.9 (3)
O6—C6—C61—C62	−157.6 (4)	C7—O7—Hf1—O1	−50.8 (3)
C3A—C6—C61—C62	20.1 (6)	C7—O7—Hf1—O4	−169.7 (3)
O6—C6—C61—C66	20.7 (5)	C1—O1—Hf1—O3	151.1 (3)
C3A—C6—C61—C66	−161.7 (4)	C1—O1—Hf1—O2	30.9 (3)
C66—C61—C62—C63	0.1 (6)	C1—O1—Hf1—O8	−126.2 (3)
C6—C61—C62—C63	178.3 (4)	C1—O1—Hf1—O6	−13.4 (4)
C61—C62—C63—C64	0.5 (7)	C1—O1—Hf1—O5	−164.2 (3)
C62—C63—C64—C65	−0.9 (7)	C1—O1—Hf1—O7	−49.2 (3)
C63—C64—C65—C66	0.8 (6)	C1—O1—Hf1—O4	89.6 (3)
C62—C61—C66—C65	−0.2 (6)	C4—O4—Hf1—O3	20.3 (3)
C6—C61—C66—C65	−178.5 (4)	C4—O4—Hf1—O2	140.5 (3)
C64—C65—C66—C61	−0.2 (6)	C4—O4—Hf1—O8	−25.4 (4)
O7—C7—C71—C72	−18.1 (5)	C4—O4—Hf1—O6	−138.2 (3)
C4A—C7—C71—C72	162.8 (4)	C4—O4—Hf1—O5	−61.2 (3)
O7—C7—C71—C76	157.2 (4)	C4—O4—Hf1—O7	−175.6 (3)
C4A—C7—C71—C76	−21.9 (6)	C4—O4—Hf1—O1	80.4 (3)

### Hydrogen-bond geometry (Å, °)

**Table:**

*D*—H···*A*	*D*—H	H···*A*	*D*···*A*	*D*—H···*A*
C43—H43···O6^i^	0.95	2.6	3.538 (5)	170.

Symmetry codes: (i) *x*, *y*+1, *z*.
